# PLAGA-PEG-PLAGA Terpolymer-Based Carriers of Herbicides for Potential Application in Environment-Friendly, Controlled Release Systems of Agrochemicals

**DOI:** 10.3390/ma13122778

**Published:** 2020-06-19

**Authors:** Kamila Lewicka, Piotr Dobrzynski, Piotr Rychter

**Affiliations:** Faculty of Science and Technology, Jan Dlugosz University in Czestochowa, 13/15 Armii Krajowej Av., 42-200 Czestochowa, Poland; lewickakamilla@gmail.com (K.L.); p.dobrzynski@ujd.edu.pl (P.D.)

**Keywords:** microsphere, PLGA, PEG, dextrin, controlled release, metazachlor, pendimethalin

## Abstract

The present study aimed to develop and prepare new polymer/herbicide formulations for their potential application in environment-friendly, controlled release systems of agrochemicals. Selected biodegradable polymers, including L-Lactide/Glycolide/PEG/Terpolymer (PLAGA-PEG-PLAGA) as well as oligosaccharide-based polymers and their blend with terpolymer, were used to prepare microspheres loaded with two soil-applied herbicides. The degradation process of the obtained polymeric microspheres was evaluated based on (1) their weight loss and surface erosion and (2) the release rate of loaded metazachlor and pendimethalin. The herbicidal effectiveness of the herbicides released to the soil from microspheres was evaluated using the European Weed Research Council (EWRC) rating scale. Moreover, the ecotoxicological effect of herbicide-loaded microspheres buried in soil on the marine bacterial species *A. fischeri* was assessed. It was found that the gradual degradation rate of microparticles led to the prolonged release of both herbicides that lasted for a few months, i.e., for the entire crop season, which is crucial in terms of agrochemical and environmental protection. Maltodextrin- and dextrin-based microspheres showed higher susceptibility to degradation than terpolymer-based microspheres. The microencapsulation of herbicides protected them from decomposition and excessive leaching into soil and maintained their activity for a longer period than that for non-immobilized herbicides. The ecotoxicological assessment on *A. fischeri* demonstrated that the proposed microsphere-encapsulated herbicides were less toxic than non-immobilized herbicides.

## 1. Introduction

Increasing global demand for food has compelled us to maximize agricultural production and has consequently led to the increased application of agrochemicals such as pesticides and fertilizers for controlling crop cultivation [1,2]. The main drawbacks of the application of conventional agrochemical formulations are the necessity for frequent treatment and the frequent use of slightly excessive amount of pesticides to provide adequate activity and the required effect of active substances. Unfortunately, as a consequence, unessential and rapid loss of these chemicals occur through various decomposition processes, including photolytic, hydrolytic or microbial degradation; volatilization; evaporation; and leaching out due to intensive rain or irrigation. As a result, the concentration of active substances in the field decreases very quickly, reaching below the minimum effective concentration, and this necessitates the repetition of treatment to maintain the biological efficacy of pesticides. In this regard, to maintain the constant, effective concentration of herbicides at the desired level, these chemicals need to be frequently applied, which leads to various environmental and health problems [1].

Nowadays, there is an increasing trend in modern agriculture towards the use of environmentally friendly release systems, especially biodegradable polymers, as carriers of active substances, which are intended to improve efficiency and limit the cost of application and minimize negative environmental effects. The active agent should be gradually released from the polymer matrix within the entire plant growth season at the level that provides optimal growth of crop plants while simultaneously killing the targeted weeds. At the same time, environmentally friendly polymeric carriers should biodegrade in soil without forming any non-degradable residues. Ideally, the active substances should be loaded into the polymer carrier only once at the beginning of the season and should release the active agent during the entire crop season through the biodegradation of the polymer [3].

Among the other, polysaccharides-based polymers are very representative, environmentally friendly carriers of active agents. Their usefulness is due to physicochemical properties like water solubility and swelling capacity, biocompatibility and biodegradability. Their non-toxic degradation products make them very popular mostly as a carrier in controlled release systems of drugs in medicine [4,5].

As gradual release systems of agrochemicals show important advantages over the conventional application schemes, many studies have been conducted on the formulation of bioactive compounds with prolonged activity, including the immobilization of herbicides through encapsulation, preparation of microspheres, etc. [6,7,8,9,10].

There are different types of carriers that have been designed and offered for the supply of medicines [11,12,13] or plant protection products and fertilizers [14,15,16,17].

The aim of this study was to prepare polymeric microspheres containing selected soil-applied herbicides, namely metazachlor and pendimethalin. Microspheres often have a multiparticulate composition and are designed to precisely reach the required target. They are frequently made from polymeric waxy or other protective materials such as natural, semi-synthetic and synthetic polymers with particle sizes ranging from 1 to 1000 μm. Many techniques are available to prepare microspheres, which provide enormous options to control optimal drug administration and to enhance their therapeutic efficacy [18,19].

The microsphere preparation method is a crucial factor in the encapsulation and release of active substances. Many factors, such as type and molecular weight of polymer, copolymer composition and solvents used, affect the size and quality of microspheres and thus may have a strong effect on the delivery rate of active substances [20,21]. Although drugs are the greatest subject of interest and are most often used for the encapsulation process for drug delivery systems, the immobilization of agrochemicals such as pesticides and fertilizers has become a challenge of modern and environmentally safe agriculture. Herbicides are one of the most widely used group of pesticides to control crop cultivation; therefore, there is an urgent need to reduce losses related to the application of excessive amounts of these active substances into soil.

Metazachlor and pendimethalin are one of the most representative and often used soil-applied herbicides worldwide. Metazachlor exhibits selective, pre-emergent effect, which inhibits the growth of plant roots; it is frequently used in rapeseed cultivation. This herbicide decomposes rather rapidly in soil with a half-life of three to nine days, and it is well soluble in water (approximately 450 mg/L). These properties may cause rapid leaching out of this herbicide during heavy rains or in an artificial irrigation process into both soil solution or groundwater, where its degradation proceeds very slowly, thereby causing accumulation in the environment (it can be found even in concentrations up to 100 μg/L) [22,23].

Pendimethalin is used in various formulations in terrestrial ecosystems. The most widely used formulation type of pendimethalin is emulsifiable concentrate because of its very low water solubility (0.3 mg/L at 20 °C). Therefore, the occurrence of this herbicide and its metabolites in soil is the result of direct application. In consequence, due to indirect exposure including evaporation, drift, leaching and runoff, it leads to contamination of surrounding aquatic ecosystem [24,25].

The application versatility of metazachlor and pendimethalin in agriculture calls for searching for new, environmentally friendly formulations with prolonged activity to minimize the negative effect on the natural environment.

## 2. Materials and Methods

### 2.1. Materials

L-Lactide and glycolide (Glaco Ltd., Beijing, China) were purified by recrystallization from ethyl acetate and was dried in a vacuum oven at room temperature. ε-caprolactone was purchased from Acros Organics (Geel, Belgium) and purified using reduced pressure vacuum distillation. Corn dextrin (dextrose equivalent DE; 9–12) and maltodextrin (DE; 12–14) were purchased from Sigma-Aldrich and used as received. Polyethylene glycol (PEG), Mw = 4600 g/mol (Aldrich Corp., Steinheim, Germany) was dried in a vacuum oven at room temperature. Catalysts, zinc acetylacetonate Zn(acac)_2_ and zirconium (IV) acetylacetonate Zr(acac)_4_ were purchased from Sigma-Aldrich, Steinheim, Germany) and used without any additional purification. Both herbicides, i.e., metazachlor and pendimethalin, were purchased from Sigma-Aldrich and used as received. Organic solvents such as methanol and chloroform were purchased and used as received (Chempur, Piekary Slaskie, Poland).

### 2.2. Synthesis of L-Lactide/Glycolide/PEG/Terpolymer (PLAGA-PEG-PLAGA)

**Scheme 1:**
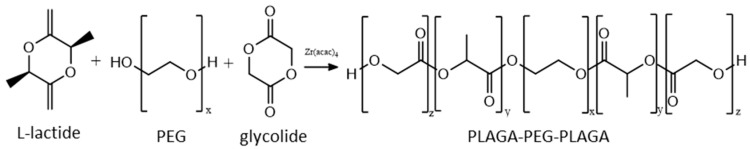
The course of terpolymer synthesis.

The terpolymers PLAGA-PEG-PLAGA was synthesized via ROP mechanism (Scheme 1) according to procedure described previously in details [26].

Briefly, polymer was synthesized via ROP mechanism. The copolymerization of L-lactide with glycolide was conducted in bulk with a zirconium (IV) acetylacetonate as catalyst with a molar ratio of 1:800 using PEG 4600 as macroinitiator. The molar ratio of L-lactide to glycolide in reaction product was 85:15 which is an accordance with initial (theoretical) calculation before reaction process. Ratio of PEG in comonomers was 20% by weight. Precipitated in cold methanol polymer had the average molecular weight about 60,000 g/mol with polydispersity index Mw/Mn = 2.8.

Polymerization was performed under argon atmosphere at 120 °C for 72 h. Obtained terpolymer was dissolved in chloroform and next precipitated in cold methanol in order to remove unreacted residues. Terpolymer was dried in vacuum at temperature 40 °C for one week to constant weight. Average molecular weight of obtained terpolymer was ~60,000 g/mol with molecular dispersion Mw/Mn = 2.8.

Detailed description of terpolymer composition was characterized previously using ^1^H and ^13^C NMR spectroscopy [26]. The weight average molecular mass of obtained PLAGA-PEG-PLAGA terpolymer was determined by GPC technique and was ~28,000 g/mol.

### 2.3. Synthesis of Dextrin and Maltodextrin Grafted with PCL (dextrin-g-PCL) and Maltodextrin-g-PCL (Maltodextrin-g-PCL) Respectively

**Figure 1:**
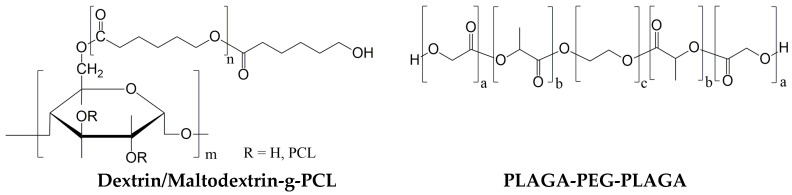
Schematic structures of synthesized polymers.

Copolymerization of dextrin or maltodextrin with ε-caprolactone (ε-CL) was described previously in details [27]. Briefly, the polymerization was carried out in bulk using calculated amount of ε-caprolactone, dextrin or maltodextrin and Zr(acac)_4_ as the catalyst (molar ratio of catalyst to ε-caprolatone was 1:600). Dextrin or maltodextrin grafted with PCL (dextrin/maltodextrin-g-PCL) were synthesized using ε-caprolactone excess in ratio 30:70. The reaction was proceeded in dried glass reactor and heated in an oil bath at 130 °C for 48 h or 72 h in an argon atmosphere with constant stirring. After the specified reaction time, unreacted ε-caprolactone and polycaprolactone were removed from bulk by extraction with chloroform. The remaining insoluble gel fraction was dried in vacuum, at temperature 40 °C. The final, dry product was washed with water to remove residues of ungrafted polysaccharides. Final product, i.e., dextrin-g-PCL or maltodextrin-g-PCL was left to dry in vacuum drier for constant mass. Conversion of ε-caprolactone grafting was above 90%. Detailed progress of the reaction including composition and degree of substitution of PCL/dextrin or maltodextrin graft were measured using nuclear magnetic resonance (^1^HNMR) spectra and was described previously in details [27]. Analogous to previously described grafting of dextrin/maltodextrin with ε-caprolactone. Calculated relative weight fraction of the glucopyranosyl ring to CL units (Rd), for grafted dextrin and maltodextrin was 70 and 38 wt%. Figure 1 demonstrates the schematic structures of synthesized terpolymer and dextrin/maltodextrin grafted with polycaprolactone.

### 2.4. Preparation of Blends Containing Terpolymer PLAGA-PEG-PLAGA and Dextrin-g-PCL or Maltodextrin-g-PCL

In brief, different aliquots of dextrin-g-PCL and maltodextrin-g-PCL solutions (10 wt.% in dry DMSO) were slowly added to 20 mL of PLAGA-PEG-PLAGA terpolymer solutions (15 wt.% in chloroform) to obtain 50 wt.% the grafted polysacharide/PLAGA-PEG blend. Blend films were prepared by pouring mixtures into Teflon dishes, drying initially for 48 h in air and then under vacuum at 40 °C to a constant mass until whole residues of solvent evaporate. The obtained two type of blends, namely PLAGA-PEG/dextrin-g-PCL and PLAGA-PEG/maltodextrin-g-PCL, in order to make them more readable, are described in further text as TER/dextrin-g-PCL and TER/maltodextrin-g-PCL blends respectively.

### 2.5. Preparation of Microspheres of PLAGA-PEG/Dextrin-g-PCL and PLAGA-PEG/Maltodextrin-g-PCL Blends Containing Herbicides

The microspheres were prepared by the precipitation of the polymer’s blends from a solution under reduced pressure ranged between 10 and 30 mBa. The PLAGA-PEG/dextrin- and maltodextrin-grafted PCL blends (1 g) were dissolved in methylene chloride (10 mL). Metazachlor (0.25 g) or pendimethalin (0.25 g) was introduced to the solution, mixed thoroughly and added dropwise into a small reactor containing 3% aqueous solution of polyvinyl alcohol (PVA, 90 mL). The reactor equipped with a stirrer and connected to a vacuum pump was placed in a bath at 23 °C. The stirring rate was 120 rpm. The rate of dropwise addition was approximately 0.3 mL/min. After an entire portion was added, the temperature of the bath was reduced to 18 °C while keeping the stirring rate constant. The vacuum pump was then switched on, reducing the pressure gradually to obtain a full vacuum. The stirring rate was decreased to approximately 50 rpm, and the process was allowed to continue under these conditions for 6–10 h until the complete evaporation of methylene chloride from the system. The obtained microspheres were washed with large amounts of redistilled water and filtered through 150-µm-diameter sieves to obtain the low range distribution of microspheres. After several hours, the upper water layer was separated, and the microspheres were dried at 25 °C under reduced pressure for 12 days to complete the evaporation of water and methylene chloride. The parameters of the abovementioned process, including type and concentration of the PVA surfactant, concentration and viscosity of the copolymer solution added, rate of dropwise addition, rate of stirring and temperature of the process, were chosen and optimized based on our previous studies dedicated to their effect on the shape and properties of the obtained microspheres [28]. The names of the obtained microspheres are analogous to the blends described above, namely PLAGA-PEG/dextrin-g-PCL and PLAGA-PEG/maltodextrin-g-PCL carriers containing herbicides, which are described in further text as TER/dextrin-g-PCL and TER/maltodextrin-g-PCL microspheres, respectively.

### 2.6. Microspheres Size Analysis

The particle size distribution of the microspheres was determined by optical microscopy. The microspheres were dispersed in water and a smear was made on a glass slide. The smear was allowed to dry in air and the size of about 300 microspheres was measured with using attached to microscope ocular and stage micrometers. Based on these observations, the percentage of microspheres with diameters in selected dimension ranges was statistically determined.

### 2.7. Determination of the Herbicides Content in the Synthesized Microspheres

The content of herbicides in the microspheres was determined by the UV–Vis spectrophotometric method using a Thermo Scientific™ Helios Gamma Spectrophotometer (Thermo Fisher, Karlsruhe, Germany). For this purpose, a weighted amount of the obtained microspheres was dissolved in chloroform, next, solution was added dropwise to cold methanol to precipitate the polymer, which was subsequently separated by centrifugation. The concentration of metazachlor or pendimethalin in remaining solution was calculated from the calibration curve prepared for both herbicides (Figure S1—Supplementary Materials).

### 2.8. Degradation Study of Microspheres

Study of microsphere degradation was conducted in three media, namely water, soil and activated sludge. After specified period of time 3-, 6- and 12-weeks samples were withdrawn from each medium and rate of degradation was evaluated by measurement of weight loss of samples and surface erosion of microspheres.

#### 2.8.1. Degradation in Distilled Water

The specified mass of microspheres was packed in membrane sacks, and then each sample was placed in a vial filled with 10 mL of distilled water at RT.

#### 2.8.2. Degradation in Activated Sludge

Activated sludge composed by total nitrogen (1.6–6.0% of dry matter); phosphorus pentoxide (P_2_O_5_, 1.5–4.0% of dry matter) and potassium oxide (K_2_O, 1–3% of dry matter) was kindly provided by treatment plant “WARTA” in Czestochowa. Total dry solids and pH of sludge ranged between 6–12% and 7.2–7.5 respectively). Each type of microspheres was placed in membrane sacks and immersed in a vial filled with 10 mL of activated sludge. Medium was replaced every two weeks. Degradation process was carried out at RT by following the microbial viability, pH analysis, sedimentation, sludge index, dry matter and visual assessment of microspheres via SEM technique.

#### 2.8.3. Degradation in Soil

Typical, sandy soil (granulometric composition—77% of sand, 16% dust and loam, organic carbon content ~1.6% and pH (KCl) = 6.6) was used for process of degradation. Soil degradation study was carried out using polypropylene membrane bags in which the microspheres were packaged, and then placed in pots with soil.

After the determined degradation time, the bags with microspheres were withdrawn from the soil or sludge, removed from the bag on the laboratory filter paper (30–50 micrometres range) and washed thoroughly (but gently to avoid damage of samples) in water, and left at RT for 3 h to maintain the same, comparable conditions for each samples. After drying, samples were weighed (Radwag, Radom, Poland).

Controlled conditions of humidity (70% field water capacity), temperature (22 ± 2 °C) and light intensity (7000 lux), under conditions imitating daytime cycles—16 h/light and 8 h/night.

### 2.9. Scanning Electron Microscopy of Microspheres

Surface evaluation of plain (non-degraded) and degraded microspheres, after specified period of their incubation time in each media, was conducted by Tescan model VEGA 3SBU scanning electron microscope (SEM) (Tescan Orsay Holding, Brno, Czech Republic). Surface measurement was conducted using accelerating voltage ranged between 1–3 kV. Scan analysis of surface was conducted at low vacuum by using the secondary electron mode and to avoid samples damage and make measurement more reliable, microspheres were not coated with a conductive layer.

### 2.10. Release Study of Herbicides into Water

All prepared microspheres loaded with 20% metazachlor and pendimethalin (TERPOLYMER, TER/dextrin and TER/maltodextrin microspheres) were immersed in closed bottles filled with 10 mL of distilled water. The medium was changed at each measurement. The samples were incubated at room temperature for 3 months. Every week, the herbicide concentration in the medium was measured. The absorbance level of released herbicides was analysed by UV–Vis spectroscopy for metazachlor and pendimethalin at λ = 228 and 238 nm respectively.

### 2.11. Determination of Herbicidal Activity Released from Microspheres

The weed growth test with metazachlor and pendimethalin as a soil-applied herbicide was performed using three widely occurring weeds, namely *Galinsoga parviflora Cav.*, *Rumex acetosa* L., and *Chenopodium album* L. Herbicidal activity of herbicides for weeds was determined by visual assessment of plant growth, damage and drying out, and was documented by digital photographs. Herbicidal activity of microspheres loaded with metazachlor and pendimethalin was evaluated in two parallel tests. First, preliminary (screening) test was aimed to find the effective dose of herbicides to be immobilized in microspheres. For this purpose, to find the effective dose of herbicides loaded in microspheres, both were tested in 3 doses, namely the dose recommended by formulation user guide as well as half and double doses of the recommended one. For metazachlor, the recommended dose of Butisan^®^400SC formulation user guide was 2.5 L/ha, which corresponds to 0.79 g/pot. In addition to the recommended dose, half and double doses were used as follows: 0.39 and 1.5 mg/pot, respectively. Pendimethalin dose recommended by the Activus^®^400SC formulation user guide was 4.0 L/ha, which corresponds to 1.26 mg per pot. Similarly, half and double doses of the recommended one were as follows: 0.63 and 2.52 mg/pot, respectively. Herbicidal effectiveness of released herbicides was evaluated on weeds after 1 month of weed growth in the presence of microspheres with immobilized herbicides in soil. After selection of the effective dose of both immobilized herbicides among the doses mentioned above, the final test of these active substances after 2 and 3 months of incubation of microspheres in soil was conducted. The effectiveness of immobilized herbicides in microspheres against the tested weeds was compared to two control series: plants grown without any herbicides and plants grown in the presence of herbicides in non-immobilized form.

Ratings were assigned on the basis of the European Weed Research Council (EWRC) scale in terms of phytointoxication as follows: 1: total plant death (100%); 2: excellent (98.0% to 99.9%); 3: very good (95.0% to 97.9%); 4: good to acceptable (90% to 94.9%); 5: moderate (82.0% to 89.9%); 6: weak (70.0% to 81.9%); 7: bad (55.0% to 69.9%); 8: very bad (30% to 54.9%); and 9: none (0.0% to 29.9%).

### 2.12. Microtox Solid Phase Assay Test

Acute toxicity of soil containing polymeric-herbicide microspheres was assessed after 1 week, 4 and 12 weeks of their incubation in soil. At the same time, for comparison, acute soil toxicity containing “pure” herbicides at similar times was determined. According to procedure, toxicological impact of the solid fraction of sample is measured on bacteria living in aqueous suspension. After regeneration of *A. fischeri* bacteria with reconstitution solution with concentration 0.01%, suspension was placed in the reagent cell/chamber of the analyser. A soil suspension was obtained via dispersion of 7 g of the soil in a solid phase diluent (35 mL of 3.5% sodium chloride solution) and was stirred afterward for 10 min with a magnetic stirrer. Bacteria (~1 × 10^6^ cell/mL per assay) were exposed for 20 min to series of various dilutions and to control (blank) of sodium chloride solution. Next, the light output of supernatants containing exposed bacteria, obtained after filtration of above suspensions, was measured with a Microtox^®^ Analyser 500. Effective concentration (EC_50_) producing biological response in half of the population habitants was determined by reduction in the light emitted by the *A. fischeri*. Statistical analysis of results was automatically computed and provided by the software of Microtox analyser.

## 3. Results and Discussion

### 3.1. Preparation of Microspheres of Terpolymer, PLAGA-PEG-PLAGA/Dextrin-g-PCL and PLAGA-PEG-PLAGA/Maltodextrin-g-PCL Blends Loaded with Herbcides

**Figure 2:**
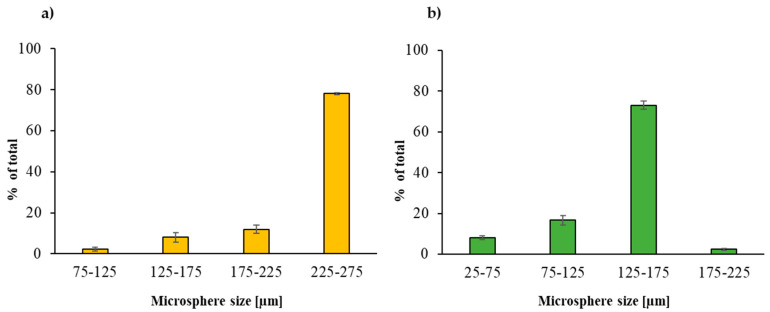
Diameters distribution of the microspheres loaded with herbicides (**a**) for TERPOLYMER and (**b**) for TER/dextrin and TER/maltodextrin.

The developed method allowed us to prepare the microspheres of relatively regular shape and narrow distribution particle size (Figure 2) with good repeatability. Microspheres with a size of 125–150 µm demonstrated the largest (nearly 80%) range among all of them.

Values of assumed and experimental percentage of herbicides in microspheres are presented in Table 1.

The reason why PLA, PLGA and PLGA-PEG were chosen to prepare microspheres containing herbicides was that these biodegradable polymers are widely used as carriers of drugs with various targets in the pharmaceutical industry and also for some agrochemicals [29,30,31,32,33,34,35,36]. As demonstrated in previous studies [26], the presence of even a small amount of PEG block in the terpolymer chain increases the hydrophilicity of the polymeric chain slightly, thereby increasing its susceptibility to hydrolytic and—unexpectedly strongly—enzymatic degradation in the soil environment. In addition, it was shown that the practical amorphism of the PLGA-PEG-PLGA terpolymers used as matrices and the lack of the phenomenon of crystalline phase formation and increase in the degree of crystallinity during all degradation processes allow for the achievement of fairly uniform progress of degradation, and thus, a relatively constant release of tested herbicides is achieved.

Unfortunately, these materials show relatively slow degradation enzymatically, and to overcome this problem, there is a need to enhance this degradation mechanism by introducing polymers that facilitate microbial action. Dextrin-g-PCL and maltodextrin-g-PCL are good candidates for this role because oligosaccharides are water soluble and easily biodegradable in the environment. Blending moderately hydrophobic terpolymer PLAGA-PEG-PLAGA with hydrophilic oligosaccharide-based polymers offers an opportunity to control the degradation rate of such materials. Enzymatic degradation of modified dextrin combined with leaching of the degradation products into medium caused significant erosion of the polymer matrix, thereby leading to the acceleration of water diffusion into the polymer matrix and allowing for the easier leaching of herbicides outside the matrix [27].

To obtain an effective system of controlled release of soil-applied herbicides, the method of microsphere formulation was used. The size of particles is a very important property of microspheres, because it affects the degradation rate, active agent loading, and initial burst release of microspheres [8]. The encapsulation yield of pendimethalin and metazachlor obtained in this study was ~75 and 85%, respectively; the difference may be due to the difference in the water solubility of these herbicides (pendimethalin: 0.33 mg/L; metazachlor: 450 mg/L) [37].

### 3.2. Degradation Study of Microspheres

**Figure 3:**
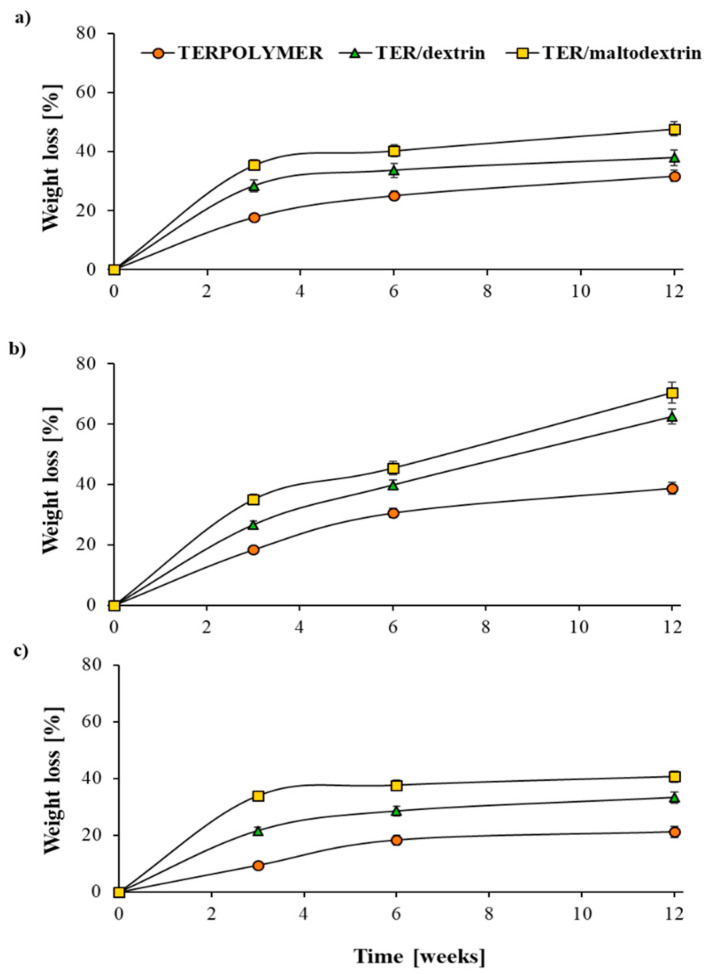
Percentage weight loss of microspheres of TERPOLYMER; TER/dextrin and TER/maltodextrin, (**a**)—in water, (**b**)—in activated sludge, (**c**)—in soil. The error bars are smaller than the symbols where they are not shown.

The degradation experiment of plain (not loaded with herbicides) microspheres was conducted in water, soil, and activated sludge. As expected, increasing weight loss of samples was observed with degradation time (Figure 3); however, it is worth noting that in each medium, the following order of weight loss of microspheres was kept: TER/maltodextrin > TER/dextrin > terpolymer. A comparison of all the used degradation media showed that the highest degree of sample weight loss was observed in activated sludge, and after 12 weeks of degradation, the weight loss was in the following order: TER/maltodextrin (~70%) > TER/dextrin (>60%) > terpolymer (almost 40%). Soil was found to be the medium in which the lowest degree of sample weight loss occurred. This is probably because of low moisture content of soil and because of limited contact with water. Consequently, limited water mobility does not allow for its distribution inside the polymer blends, and the dissolution of low-molecular-weight degradation products of blends is reduced.

This aspect is highly advantageous in terms of agrochemical and environmental protection because it allows for the slow degradation of the microspheres and thus gradual release of active substances such as herbicides outside the microspheres for several weeks. The rapid weight loss of samples at the initial phase of the degradation process was due to the release of polymer degradation products into the medium. Regardless of the medium used, microspheres of TER/maltodextrin were found to be the most susceptible to degradation. The rate of degradation of these microspheres was almost 50% after three months; thus, it can be assumed that the degradation of the remaining carriers will not take more than one year. The highest weight loss of TER/maltodextrin in comparison to other microspheres is probably due to its highest susceptibility to water, which is associated with the presence of low-molecular-weight maltodextrin that is easily soluble in water. In contrast, among the tested microspheres, terpolymer as the most hydrophobic compound was found to be the most resistant material to degradation. Therefore, TER/maltodextrin mostly meets the criteria of controlled release systems of herbicides because microspheres (after release of herbicides) will be totally degraded and disappear from soil within a growing season; moreover, they not only serve as a source of available carbon for microorganisms but also improve the soil condition.

### 3.3. Scanning Electron Microscopy (SEM) of Microspheres

The obtained microspheres of TER/dextrin and TER/maltodextrin were non-porous, smooth surfaced and spherical in structure under a scanning electron microscope, with a mean particle size ranging from 125 to 175 µm. For comparison, microspheres prepared from terpolymer were larger and had an average particle size of 225–275 µm.

**Figure 4:**
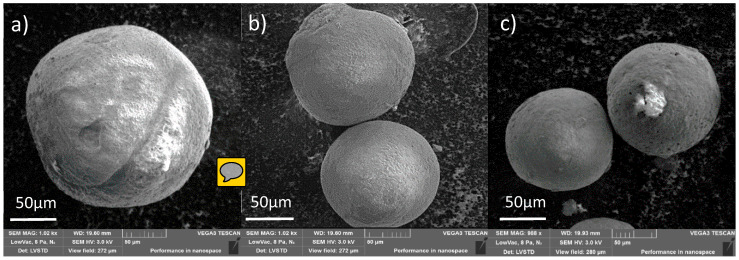
Scanning electron microscopy (SEM) images of obtained microspheres: (**a**) terpolymer, (**b**) TER/dextrin (**c**) TER/maltodextrin (magnification × 1000).

**Figure 5:**
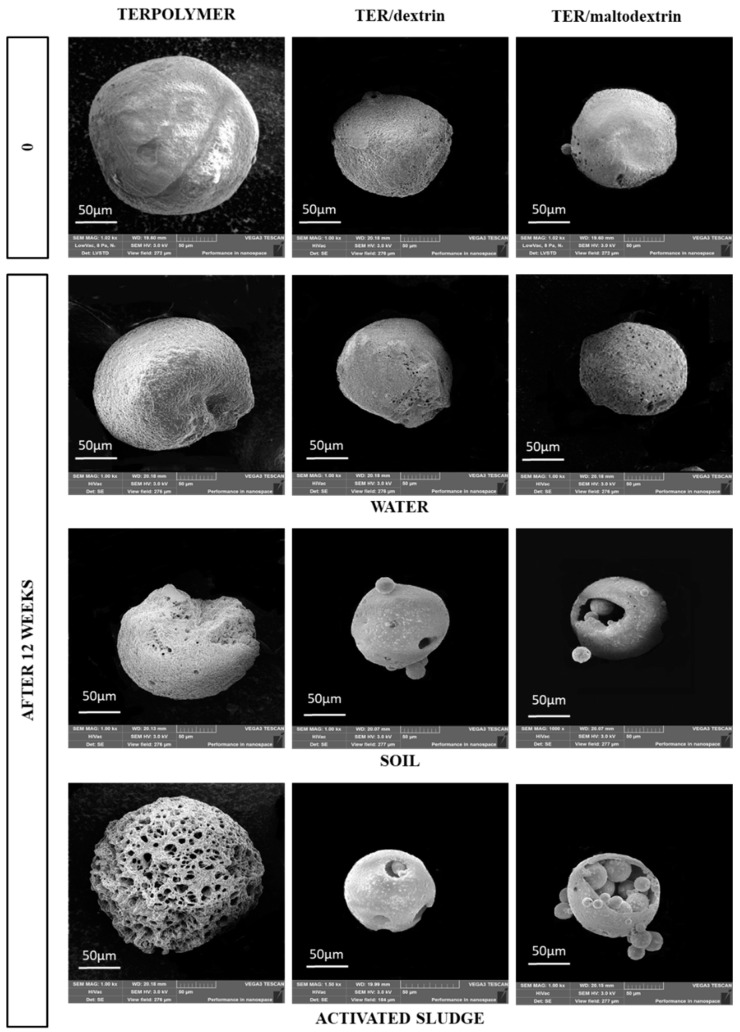
Deterioration of the microspheres surface after each period of exposure to the degradation medium, scanning electron microscopy (SEM) (magnification × 1000).

The surface erosion of microspheres incubated in water, soil and activated sludge after the specified period of time was monitored by scanning electron microscopy (SEM). The initial images of all microspheres demonstrated that the surface of plain terpolymer had the most homogenous nature when compared with the surface of other blends containing dextrin- or maltodextrin-based polymers (Figure 4). The analysis of SEM images showed an ambiguous effect of microsphere surface erosion. The degree of surface erosion was dependent on the medium in which microspheres were incubated. As expected, activated sludge had the strongest impact on surface degradation of all microspheres. A comparison of all used degradation media showed that activated sludge possessed the highest microbial activity and the most variable chemical composition, including nitrogen, potassium and phosphorus, which facilitated microbial activity [29]. This is reflected in gradual increasing surface erosion with the increase in incubation time. For pure water, the hydrolytic degradation mechanism dominates due to the easy mobility of water molecules within the polymer matrix, while the enzymatic degradation combined with hydrolysis is a crucial mechanism of sample disintegration in activated sludge and soil. For maltodextrin- and dextrin-based microspheres, more heterogenous changes on the surface, including larger cracks, splits and cleavages, were observed. The analysis of SEM images revealed that the obtained microspheres containing maltodextrin or dextrin comprise lower size microspheres (Figure 5).

Ongoing surface erosion of all samples and their weight loss in each medium confirm both enzymatic and hydrolytic degradation (deterioration) of all microspheres within several weeks. This time period of microsphere degradation allows for the prolonged release of any type of active agent, including herbicides, loaded inside the microspheres.

A comparison of surface erosion of the tested microspheres with that of their analogous films showed that regardless of the degradation medium, the weight loss of both microspheres and films was similar after 12 weeks for each carrier (TERPOLYMER, TER/dextrin and TER/maltodextrin).

PLA and PLAGA microspheres are already reported in literature to be easily degraded due to high surface area to volume ratio (i.e., higher accessibility to water), which enhances the diffusion of active agent through the polymer coat [8].

### 3.4. Release Study of Herbicides into Water

**Figure 6:**
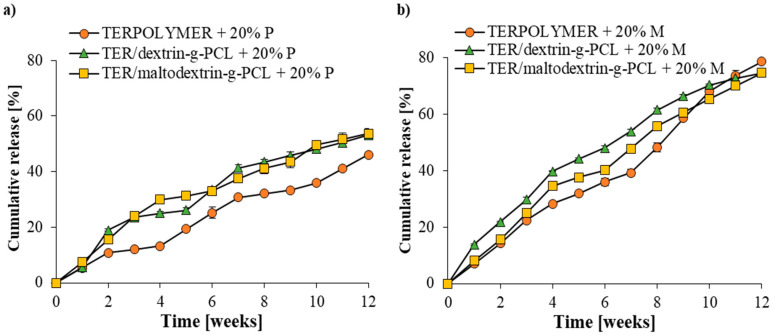
Cumulative release of pendimethalin (**a**) and metazachlor (**b**) from microspheres into water. The error bars are smaller than the symbols where they are not shown.

The release rate of the loaded herbicides metazachlor and pendimethalin (20% by weight of polymer carrier) in each type of microsphere is shown in Figure 6. The amount of metazachlor and pendimethalin released from the microsphere was almost 80% and 60% within three months; thus, it is clear that the release rate is correlated with the degradation of the microsphere surface as described in the previous section. Weight loss associated with surface erosion allowed for the release of both herbicides from the polymer matrix. A higher amount of release was noted for metazachlor, most probably because of its much higher solubility in water than that of pendimethalin. Furthermore, the lowest amount (~50%) of pendimethalin released from terpolymer without dextrin or maltodextrin was because this herbicide has very low solubility in water and terpolymer is relatively more hydrophobic than blends with grafted maltodextrin or dextrin-based polymers.

A comparison of release of both herbicides from all microspheres with the release rate of the same herbicides from polymer films estimated in our previous study showed few differences. The release rate of metazachlor and pendimethalin from microspheres was 79% and 46%, respectively, while their release from the same terpolymer films was 54% and 29% for metazachlor and pendimethalin, respectively [26]. For polymer films formed from TER/dextrin, the release rate was 67% and 45% for metazachlor and pendimethalin, respectively, while for polymer films formed from TER/maltodextrin, the release rate was 68% and 49% for metazachlor and pendimethalin, respectively [27].

It is worth noting that by using the proposed microspheres, the undesirable burst effect that often occurs during the release process was significantly reduced. The slow degradation of all the used polymeric microspheres allowed for the gradual release of herbicides even at the initial stage of incubation process. This is a great advantage in terms of environmental protection, because the initial high dose of the released herbicide does not contaminate the soil and thus minimizes its losses.

In previous experiments, the burst effect of both tested herbicides was observed when released from polymeric films. The burst release mechanism depends on the nature of the active substance delivery system. Rapid release may be due to the high specific surface that is in contact with external media; in this case, it was activated sludge, soil and water. Polymeric herbicide films made by solvent casting can be dispersed or localized on the surface of a support, and when they come in contact with the medium, it results in high release of the herbicide. The cumulative release of metazachlor and pendimethalin from the microspheres showed that the plateau phase was not reached. The release of both herbicides was still in the growth phase, even after 12 weeks.

**Figure 7:**
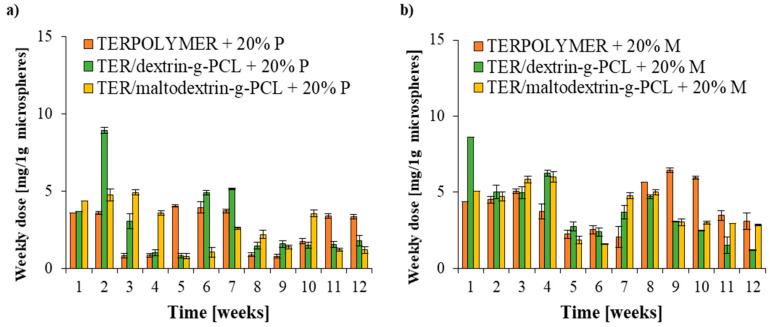
Weekly dose release of (**a**) pendimethalin and (**b**) metazachlor from microspheres into water.

The average weekly released doses of these herbicides (Figure 7) over the observed time were almost the same. The graphs show that the polymer formulation in the form of microspheres improves the release profile. The weekly release rates of both metazachlor and pendimethalin were constant over a period of 12 weeks.

Although controlled release systems of agrochemicals have currently become a challenge for modern and environmentally safe agriculture, very few studies have been conducted on polymer/herbicide formulation with prolonged activity. In particular, there are almost no studies on microencapsulation formulation and the release of soil-applied herbicides, including metazachlor and pendimethalin [8,37]. Stloukal et al. (2012) successfully encapsulated metazachlor using low-molecular-weight poly(lactic acid) (PLA) [8]. In their study, three series of polymer micro- and submicroparticles with varying sizes were prepared from low-molecular-weight PLA by the oil-in-water solvent evaporation technique and were loaded with various initial amounts of the herbicide metazachlor. The release rate of the herbicide increased with its loading. The authors proved that the preparation of a controlled release formulation of the herbicide metazachlor with relatively high-water solubility based on biodegradable PLA microparticles was possible in principle.

Włodarczyk (2011) investigated alginate-based hydrogel microcapsules [38]. The author examined the kinetics of releasing metazachlor from the matrix at various temperatures under laboratory conditions. The results revealed that the concentration of metazachlor released from the hydrogel capsules to the aquatic environment grows exponentially over time and depends on the type and temperature of the aquatic environment.

Few attempts were devoted to the encapsulation of pendimethalin using PVA, polyvinylpyrrolidone, hydroxyalkylcellulose, phenol formaldehyde, gelatine, etc. [39,40,41]. These studies assessed the influence of various methods on the encapsulation efficiency of pendimethalin.

Kumar and Chinnamuthu [42] conducted a study on polyethylene glycol/pendimethalin formulation and reported that the herbicide immobilized inside the polymer was well protected from the environmental factors and that the gradual release of pendimethalin was moisture dependent.

### 3.5. Determination of Herbicidal Activity Released from Microspheres

**Figure 8:**
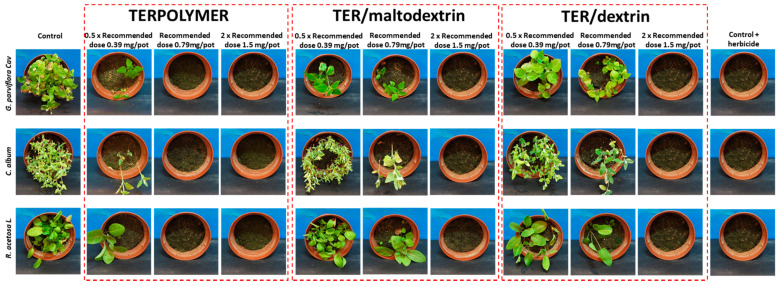
Herbicidal activity assessment of released metazachlor from microspheres after one month of incubation of microspheres in soil.

**Figure 9:**
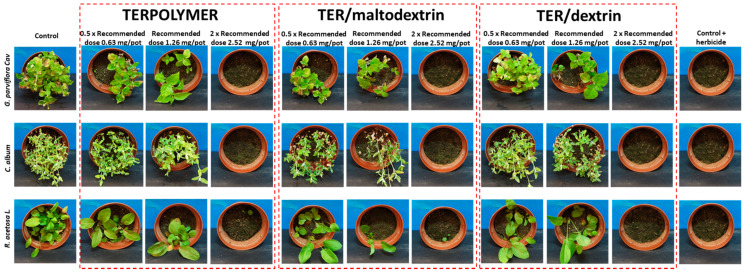
Herbicidal activity assessment of released pendimethalin from microspheres after one month of incubation of microspheres in soil.

The visual effect of metazachlor and pendimethalin released from all the used microspheres on the examined species of weeds was evaluated after one month of their growth in soil and is presented in Figure 8 and Figure 9.

Ratings were assigned using scales from the EWRC and are presented in Table 2 and Table 3 for metazachlor and pendimethalin, respectively. The obtained results for the herbicidal effectiveness of the tested microspheres showed that the inhibition effect on weeds was dependent on the amount of herbicide loaded inside the microspheres. A double dose of the recommended one by the manufacturer was found to be effective for both herbicides against all the tested weeds. Among the recommended doses, metazachlor was effective when immobilized in terpolymer and TER/dextrin microspheres. The dose of pendimethalin recommended by the manufacturer was effective only when it was immobilized in TER/maltodextrin microspheres. The results of this screening test demonstrated that although the doses of both herbicides recommended by the manufacturer were effective when used in the conventional way (non-immobilized), they were not sufficient when the herbicides were immobilized inside the proposed microspheres. We found that a double dose is recommended for using immobilized form of microsphere-encapsulated herbicides. Only these doses ensured sufficient release of both herbicides from microspheres and provided their effectiveness against the tested weeds.

On the basis of these results, pots with a double dose of herbicides were chosen for further experiments.

**Figure 10:**
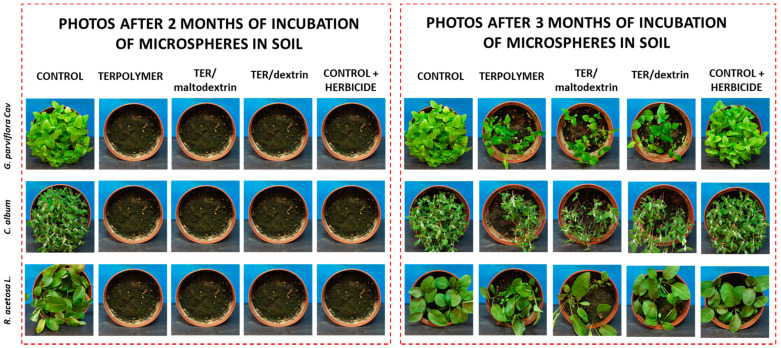
Herbicidal activity assessment of released metazachlor loaded into microspheres in double recommended dose. Seeds of each plant were sown after two and three months of incubation of microspheres in soil.

**Figure 11:**
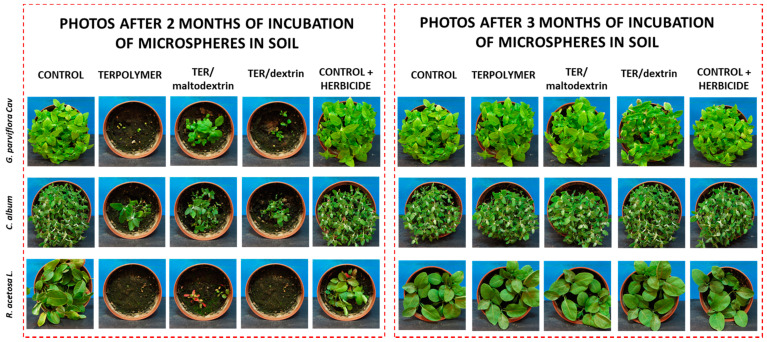
Herbicidal activity assessment of released pendimethalin loaded into microspheres in double recommended dose. Seeds of each plant were sown after two and three months of incubation of microspheres in soil.

Because after one month of seed sowing, there were no germinated plants in the pots with buried microspheres loaded with double dose of herbicides, new seeds were sown to evaluate the herbicidal activity after two months. The same treatment was performed after the second month to finally evaluate the effectiveness of released herbicides from microspheres buried for three months in soil (Figure 10 and Figure 11).

Immobilized metazachlor in all types of microspheres totally inhibited seed germination after two months of its presence in soil. The same effect was observed for the non-immobilized herbicide. However, it is worth noting that the herbicidal activity of metazachlor evaluated according to the EWRC scale after three months of incubation of microspheres in soil (Table 4 and Table 5) revealed that the encapsulated active substance was more effective against the tested weeds (EWRC: 6 in rating scale against *G. parviflora* and *C. album*) than the non-immobilized one (EWRC: 9 in rating scale against all the tested weed species). The reduced herbicidal activity of metazachlor used in the classical, non-immobilized form is due to its relatively short time of half-life (DT_50_) in soil; however, when used in microsphere-encapsulated form, it is protected against external conditions of the soil environment, which retains its activity for a longer period of time. Considering these results, it is possible to control the release rate of herbicides from the used microspheres by changing the dose of the active substance loaded inside the microspheres. Instead of multiple applications of the classical herbicidal formulation (non-coated), one-time immobilization of higher dose of metazachlor in the proposed microspheres (higher than the tested dose in this study, e.g., triple dose) may release this herbicide throughout the entire plant growth season and thus effectively inhibit the weed growth. This is highly advantageous in terms of environmental protection, and such systems comprising encapsulated herbicides should be considered for further, more comprehensive studies in real field conditions.

The encapsulated pendimethalin demonstrated its herbicidal activity after one and two months of incubation of all microspheres in soil. It should be highlighted here that after two months, pendimethalin released from all microspheres was much more effective against the tested weeds than the non-immobilized herbicide. This finding confirms the protective features of the used microspheres from external soil environment that causes rapid decomposition or leaching of the enclosed herbicide.

### 3.6. Microtox Assay Test

**Figure 12:**
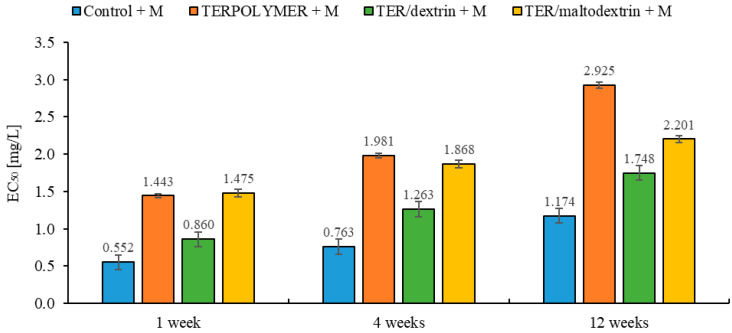
EC_50_ values of tested soil with microspheres (TERPOLYMER, TER/dextrin, TER/maltodextrin) loaded with metazachlor measured during exposure to the *A. fischeri*.

**Figure 13:**
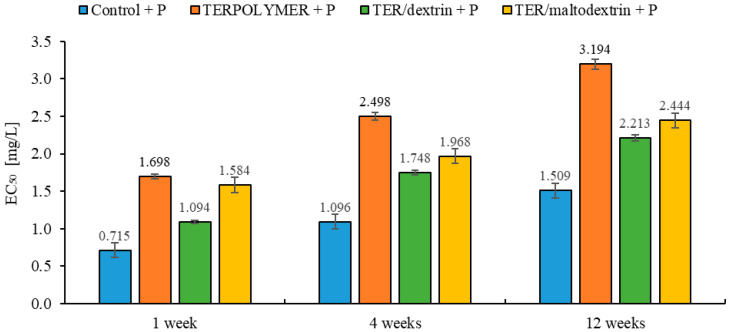
EC_50_ values of tested soil with microspheres (TERPOLYMER, TER/dextrin, TER/maltodextrin) loaded with pendimethalin measured during exposure to the *A. fischeri*.

The luminescent marine bacteria *A. fischeri* test is one of the most commonly used biotests. Values of EC_50_ calculated by the Microtox Analyzer software are shown in Table 6 and plotted in Figure 12 for metazachlor and Table 7 and Figure 13 for pendimethalin. The results of toxicity assessment using *A. fischeri* as the test organism showed different toxicity results.

As expected, the highest toxicity against *A. fischeri* was found for soil contaminated with both non-coated herbicides.

The effective concentration value (EC_50_) measured by the Microtox Analyzer software after one week of the presence of microspheres in soil demonstrated the following order of sample toxicity: soil loaded with non-coated herbicides > soil with TER/dextrin > soil with TER/maltodextrin > soil with terpolymer. During the time of experiment, all EC_50_ values increased in the same order. This implies that the toxicity of all formulations decreased in time when incubated in soil. Among the microspheres, regardless of the encapsulated herbicide, terpolymer was found to be the least toxic to *A. fischeri* at each time period as compared to maltodextrin- and dextrin-based microspheres. This is probably because among the tested polymeric coatings, terpolymer is the most hydrophobic compound and is not as easily disintegrated as grafted oligosaccharide-based microspheres. Thus, both herbicides are released much more slowly, which reflects on toxicity to *A. fischeri*.

TER/dextrin and TER/maltodextrin microspheres containing both herbicides demonstrated a higher negative effect on the tested bacterial species due to the faster release of active substances caused by rapid surface erosion of microspheres as described earlier. A comparison of both maltodextrin- and dextrin-based microspheres showed that the latter demonstrated higher toxicity against *A. fischeri.* This may be caused by the fact that dextrin includes a wider spectrum of oligosaccharides with various molecular weights and water solubility than maltodextrin and this may cause a negative effect on the tested microorganisms.

The effectiveness of metazachlor and pendimethalin on the selected plant species, including hoe nightshade (*Solanum physalifolium*), goosefoot (*Chenopodium album*) and annual mercury (*Mercurialis annua*), was studied by Jursik et al. (2019) [43]. The authors demonstrated the ambiguous toxicity of both herbicides, which was dependent on irrigation intensity and plant specimen. Pendimethalin was much more sensitive on *C. album* and *M. annua*, while metazachlor was much more toxic against *S. physalifolium.* Mohr et al. showed strong negative effects of both herbicides on the tested macrophytes *Potamogeton natans*, *Myriophyllum verticillatum* and filamentous green algae [24,44].

Bražėnaitė et al. [45] investigated the bioavailability and toxicity of pendimethalin to three species of aquatic microorganisms: green microalgae *Selenastrum capricornutum*, ciliate protozoa *Tetrahymena thermophile* and luminescent bacteria *Vibrio fischeri*; they demonstrated that the first species was the most sensitive to pendimethalin (EC_50_: 52 μg/L). For the remaining two species, the EC_50_ values were above the water solubility level of this compound (0.3 mg/L).

The obtained results showed that the proposed composition of microspheres is less toxic to the tested bacterial species *A. fischeri* than non-immobilised herbicides in all the tested periods. This is highly advantageous in terms of environmental protection because these microspheres enable the controlled release of herbicides for several weeks and simultaneously reduce the ecotoxicological effect of immobilized active substances. These prolonged release systems of agrochemicals should significantly improve the quality of intensively cultivated agricultural areas, especially the population of groundwater microorganisms. Furthermore, these systems have an economical benefit related to the reduction in the amount of herbicide usage and number of treatments in a plant growth season [46,47,48].

## 4. Conclusions

This paper is a part of series reports devoted to potential application of terpolymer-based polymers towards agricultural and agrochemical purposes. In the first paper of this series, we focused on the synthesis and characterization of PLAGA-PEG-PLAGA terpolymer containing 10 and 20% of polyethylene glycol with various molecular weight [26]. The degradation study of prepared polymeric films in various media like activated sludge, soil and water as well as the evaluation of release profiles of soil-applied herbicides (metazachlor and pendimethalin) from these films, allowed for the selection of the film with the best polymeric composition from an agricultural point of view. Among the tested terpolymers, PLAGA-PEG-PLAGA containing PEG with Mw = 4600 g/mol was found to be the best for further investigation towards the preparation of herbicidal formulation with prolonged activity. It was found that even 10% content of PEG in terpolymer facilitates its enzymatic degradation, however it is worth noting that the results were still not satisfactory, because the degradation rate of polymer and release of herbicides could be better. To overcome this problem and make terpolymer even more facilitative to microbial attack and faster enzymatic degradation, our second attempt dealt with the preparation of blends of grafted dextrin/maltodextrin with terpolymer PLAGA-PEG-PLAGA. We found that by blending olisacharide-based polymers (dextrin or maltodextrin grafted with PCL) with the obtained terpolymer, it is possible to improve the effectiveness of herbicide release [27]. Both the type and content of oligasacharides in blend played a role in degradation of the polymeric matrix, releasing—at the same time—herbicides of almost 90% of the total load within a few months, i.e., the whole plant growth season. Based on the obtained results and knowing that the release of herbicides loaded above polymeric films is dependent on polymer and blend composition and dose of loaded herbicides in the polymeric matrix, we decided to evaluate, in this study, the impact of the type of formulation on the effectiveness of proposed herbicides with prolonged activity. We used the same polymers as described above for the preparation of microspheres instead of polymeric films.

In this work, we demonstrated that by using three types of polymeric materials, namely, PLAGA-PEG-PLAGA (terpolymer), PLAGA-PEG/dextrin-g-PCL (TER/dextrin) and PLAGA-PEG/maltodextrin-g-PCL (TER/maltodextrin), it is possible to obtain microspheres with low diameter distribution that can be loaded with herbicides (or any other biologically active substances). The obtained results related to the degradation of these microparticles proved that gradual weight loss and surface erosion enabled the prolonged release of the active substance for a few months, which is required from the agrochemical point of view. The controlled degradation rate of microspheres associated with the gradual release of both tested soil-applied herbicides (metazachlor and pendimethalin) provided the effective protection of crop plants against the tested weeds for two to three months after introduction into the soil. Among the tested microspheres, the fastest degradation and release rate was found for oligosaccharide-based polymers when compared with PLAGA-PEG-PLAGA terpolymer. This is undoubtedly caused by the fact that maltodextrin- and dextrin-based microspheres are much more hydrophilic and thus facilitate enzymatic degradation of materials through the action of microorganisms living in soil. The obtained results indicate that by changing the ratio of blends, it is possible to design microspheres that can release herbicides throughout the plant growth season. We found that microencapsulation protects herbicides from decomposition and excessive leaching into the soil environment, thus maintaining their activity for a longer period of time than that of non-immobilized herbicides. Moreover, the ecotoxicological assessment on the bacterial species *A. fischeri* proved that the proposed microspheres were less toxic than the classical, non-coated herbicidal formulation. We believe that to limit environmental contamination with pesticides, such non-toxic controlled release systems with prolonged activity of agrochemicals would be beneficial for the development of sustainable agriculture, and thus, more comprehensive studies in real field conditions should be undertaken in the future.

## Figures and Tables

**Table 1:** Determination of the metazachlor/pendimethalin content in microspheres.

Sample	Herbicides	M^0^_H_/M_P_ [%]	M^e^_H_/M_P_ [%]
TERPOLYMER	Metazachlor	20	16
	Pendimethalin	20	15
TER/maltodextrin	Metazachlor	20	17
	Pendimethalin	20	17
TER/dextrin	Metazachlor	20	16
	Pendimethalin	20	15

M^0^_H_/M_P_—assumed percentage of herbicides in microspheres. M^e^_H_/M_P_—experimental percentage of herbicides in microspheres.

**Table 2:** Herbicidal activity assessment of metazachlor released from microspheres after one month of their incubation in soil (European Weed Research Council (EWRC) scale. Herbicides were loaded in three doses: half of recommended (0.39 mg/pot), recommended (0.79 mg/pot) and double dose (1.5 mg/pot).

		TERPOLYMER			TER/Maltodextrin			TER/Dextrin			
Weeds	Control	0.39 mg/pot	0.79 mg/pot	1.5 mg/pot	0.39 mg/pot	0.79 mg/pot	1.5 mg/pot	0.39 mg/pot	0.79 mg/pot	1.5 mg/pot	Control + Herbicide
*G. parviflora Cav.*	−	3	1	1	5	4	1	8	7	1	1
*C. album L*.	−	3	1	1	8	3	1	9	6	1	1
*R. acetosa* L.	−	3	1	1	9	6	1	8	5	1	1

**Table 3:** Herbicidal activity assessment of pendimethalin released from microspheres after one month of their incubation in soil (EWRC scale). Herbicides were loaded in three doses: half of recommended (0.63 mg/pot), recommended (1.26 mg/pot) and double dose (2.52 mg/pot).

		TERPOLYMER			TER/Maltodextrin			TER/Dextrin			
Weeds	Control	0.63 mg/pot	1.26 mg/pot	2.52 mg/pot	0.63 mg/pot	1.26 mg/pot	2.52 mg/pot	0.63 mg/pot	1.26 mg/pot	2.52 mg/pot	Control + Herbicide
*G. parviflora Cav.*	−	7	6	1	8	7	1	9	4	1	1
*C. album L*.	−	9	8	1	9	5	1	9	8	1	1
*R. acetosa* L.	−	7	6	1	8	3	2	8	7	1	1

**Table 4:** Herbicidal activity assessment of released metazachlor loaded into microspheres in double recommended dose (EWRC scale).

		2 Months after Incubation of Microspheres in Soil				3 Months after Incubation of Microspheres in Soil			
Weeds	Control	TERPOLYMER	TER/maltodextrin	TER/dextrin	Control + Herbicide	TERPOLYMER	TER/maltodextrin	TER/dextrin	Control + Herbicide
*G. parviflora Cav.*	−	1	1	1	1	6	6	6	9
*C. album L*.	−	1	1	1	1	6	6	6	9
*R. acetosa* L.	−	1	1	1	1	8	7	8	9

**Table 5:** Herbicidal activity assessment of released pendimethalin loaded into microspheres in double recommended dose (EWRC scale).

		2 Months after Incubation of Microspheres in Soil				3 Months after Incubation of Microspheres in Soil			
Weeds	Control	TERPOLYMER	TER/maltodextrin	TER/dextrin	Control + Herbicide	TERPOLYMER	TER/maltodextrin	TER/dextrin	Control + Herbicide
*G. parviflora Cav.*	−	3	6	5	9	9	9	9	9
*C. album L*.	−	4	6	4	9	9	9	9	9
*R. acetosa* L.	−	1	3	1	9	9	9	9	9

Note Table: 1 = total plant death (100%); 2 = excellent (98.0% to 99.9%); 3 = very good (95.0% to 97.9%); 4 = good to acceptable (90% to 94.9%); 5 = moderate (82.0% to 89.9%); 6 = weak (70.0% to 81.9%); 7 = bad (55.0% to 69.9%); 8 = very bad (30% to 54.9%); and 9 = none (0.0% to 29.9%).

**Table 6:** Microtox^®^ EC_50_ values (mg/L) of exposure of the luminescent marine bacteria *A. fischeri* to tested soil with microspheres (TERPOLYMER, TER/dextrin, TER/maltodextrin) with metazachlor respective to 95% confidence limits (in brackets) obtained in the fit of the data.

1 Week		4 Weeks		12 Weeks	
EC_50_ (Lower Limit, Upper Limit [mg/L])	Coefficient of Determination (R^2^)	EC_50_ (Lower Limit, Upper Limit [mg/L])	Coefficient of Determination (R^2^)	EC_50_ (Lower Limit, Upper Limit [mg/L])	Coefficient of Determination (R^2^)
**Control + M**					
0.552 (0.450; 0.677)	0.957	0.763 (0.647; 0.899)	0.973	1.174 (1.025; 1.344)	0.978
**TERPOLYMER + M**					
1.443 (1.138; 1.831)	0.936	1.981 (1.572; 2.496)	0.947	2.925 (1.963; 4.357)	0.845
**TER/dextrin + M**					
0.860 (0.719; 1.029)	0.965	1.263 (1.089; 1.466)	0.978	1.748 (1.529; 1.999)	0.977
**TER/maltodextrin + M**					
1.475 (1.164; 1.869)	0.926	1.868 (1.315; 2.652)	0.823	2.201 (1.860; 2.605)	0.958

**Table 7:** Microtox^®^ EC_50_ values (mg/L) of exposure of the luminescent marine bacteria *A. fischeri* to tested soil with microspheres (TERPOLYMER, TER/dextrin, TER/maltodextrin) with pendimethalin respective to 95% confidence limits (in brackets) obtained in the fit of the data.

1 week		4 weeks		12 weeks	
EC_50_ (Lower Limit, Upper Limit [mg/L])	Coefficient of Determination (R^2^)	EC_50_ (Lower Limit, Upper Limit [mg/L])	Coefficient of Determination (R^2^)	EC_50_ (Lower Limit, Upper Limit [mg/L])	Coefficient of Determination (R^2^)
**Control + P**					
0.715 (0.589; 0.868)	0.961	1.096 (0.971; 1.237)	0.985	1.509 (1.408; 1.618)	0.995
**TERPOLYMER + P**					
1.698 (1.362; 2.116)	0.949	2.498 (1.926; 3.239)	0.933	3.194 (2.423; 4.211)	0.906
**TER/dextrin + P**					
1.094 (0.937; 1.278)	0.973	1.748 (1.568; 1.949)	0.988	2.213 (1.950; 2.512)	0.985
**TER/maltodextrin + P**					
1.584 (1.322; 1.897)	0.965	1.968 (1.648; 2.349)	0.963	2.444 (1.978; 3.019)	0.939

## Supplementary Materials

The following are available online at https://www.mdpi.com/1996-1944/13/12/2778/s1, Figure S1: Calibration curve of herbicides solution standards.
