# Supplementary material for: PLAGA-PEG-PLAGA Terpolymer-Based Carriers of Herbicides for Potential Application in Environment-Friendly, Controlled Release Systems of Agrochemicals
Source: Materials (Basel). 2020 Jun 19;13(12):2778. doi: 10.3390/ma13122778 (PMC7345335; doi:10.3390/ma13122778)
Supplement: Supplementary file 1 [file materials-13-02778-s001.pdf]

materials

Supplementary Materials

PLAGA-PEG-PLAGA Terpolymer-Based Carriers of Herbicides for Potential Application in Environment-Friendly, Controlled Release Systems of Agrochemicals

Kamila Lewicka, Piotr Dobrzynski and Piotr Rychter *

Faculty of Science and Technology, Jan Dlugosz University in Czestochowa, 13/15 Armii Krajowej Av., 42-200 Czestochowa, Poland; lewickakamilla@gmail.com (K.L.); p.dobrzynski@ujd.edu.pl (P.D.)

* Correspondence: p.rychter@ujd.edu.pl

Received: 29 May 2020; Accepted: 17 June 2020; Published: date

Preparation of herbicides calibration curve

The calibration curve was prepared using a series of samples of each herbicide concentration solutions (herbicide standards in water). A curve was fitted to the data, and the resulting equation was used to convert readings of the unknown samples into concentrations. The unknown concentration of released herbicide was measured mathematically by solving the equation for concentration as a function of signal.

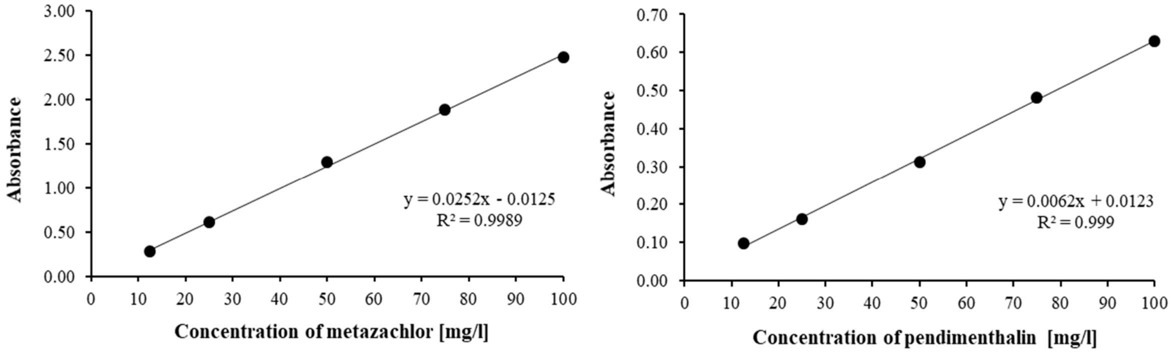

Figure S1. Calibration curve of herbicides solution standards.

© 2020 by the authors. Submitted for possible open access publication under the terms and conditions of the Creative Commons Attribution (CC BY) license (http://creativecommons.org/licenses/by/4.0/).
